# Prevalence of Unintended Pregnancy and Associated Factors Among Women Enrolled in the Support-Moms Study in Southwestern Uganda: A Cross-Sectional Analysis of Baseline Data

**DOI:** 10.7759/cureus.107426

**Published:** 2026-04-20

**Authors:** Julius Mugisha, Stuart Turanzomwe, Mugyenyi R Godfrey, Angella Musiimenta, Micheal Kanyesigye, Henry Ochola, Elly Atuhumuza, Esther C Atukunda

**Affiliations:** 1 Obstetrics and Gynaecology, Mbarara University of Science and Technology, Mbarara, UGA; 2 Obstetrics and Gynaecology, Kabale University, Mbarara, UGA; 3 Research and Innovations, Angels Compassion, Mbarara, UGA; 4 Information Technology, Mbarara University of Science and Technology, Mbarara, UGA; 5 Public Health, Mbarara University of Science and Technology, Mbarara, UGA; 6 Obstetrics and Technology, Mbarara University of Science and Technology, Mbarara, UGA; 7 Pharmacy, Mbarara University of Science and Technology, Mbarara, UGA

**Keywords:** mistimed pregnancy, pregnancy desire, southwestern uganda, unintended pregnancy, unwanted pregnancy

## Abstract

Unintended pregnancies and unmet need for contraception continue to be a public health concern in Sub-Saharan African countries. This potentially contributes to the high maternal mortality rate in these countries. This study highlights the burden and the context-specific factors associated with unintended pregnancy in Southwestern Uganda. This was a secondary analysis of baseline data from pregnant women enrolled in the Support-Moms trial, which was a community-based randomized controlled trial. The Support-Moms trial recruited 824 pregnant women who had not started antenatal care visits, and these were randomly assigned (1:1) into two groups: the control group, which received standard of care, and the intervention group that received health information messages in the form of Short Messaging Service (SMS) or audio from the Support-Moms app. Participants were recruited from Mbarara City, Mbarara District, and Mitooma District. Enrolled women completed an interviewer-administered questionnaire to collect data on sociodemographic characteristics, reproductive history, prior birth and pregnancy outcomes, household food security, and pregnancy intention. Women who had unwanted or mistimed pregnancies were collectively regarded as having an unintended pregnancy. Data analysis was done using Stata version 19 (StataCorp LLC, College Station, TX), and a modified Poisson regression analysis was employed to document the factors associated with unintended pregnancy. Statistical significance was set at a p-value < 0.05. To assess the prevalence of unintended pregnancy and the associated factors, a total of 818 women were included in the final analysis. The median age was 27 years. Overall, 296 (36.2%) women reported an unintended pregnancy. In multivariable analysis, moderate (adjusted prevalence ratio (aPR) = 1.55; 95% CI: 1.02-2.36) and severe food insecurity (aPR = 1.96; 95% CI: 1.25-3.06), grand multigravidity (aPR = 1.64; 95% CI: 1.11-2.41), and lack of awareness of the partner’s desired number of children (aPR = 1.22; 95% CI: 1.01-1.47) were independently associated with unintended pregnancy. Over one-third of pregnant women in the Support-Moms trial, Southwestern Uganda, had unintended pregnancy, largely driven by food insecurity, grand multigravidity, and inadequate partner communication. Context-specific interventions are critical to mitigating this high burden.

## Introduction

Unintended pregnancy, defined as pregnancies that were mistimed or unwanted at the time of conception, remains a key global public health issue affecting an estimated 121 million women each year [1]. The burden of unintended pregnancy in Sub-Saharan Africa remains considerably higher, with a reported incidence rate of 70-90 unintended pregnancies per 1,000 women of reproductive age compared to the global average of approximately 64 unintended pregnancies per 1,000 women of reproductive age [1,2]. Overall, nearly one-third of pregnancies in the region are unintended, key drivers being persistent unmet need for modern contraception, early initiation of sexual activity, entrenched gender inequalities, widespread poverty, and under-resourced health systems [3,4]. Unintended pregnancy remains one of the common causes of induced abortions, one of the common direct causes of maternal mortality [5,6].

In Uganda, unintended pregnancy remains a common occurrence despite improvements in access to and uptake of contraceptive services, with rates of up to 145 unintended pregnancies per 1000 women aged 15-49 years. Evidence from facility-based and longitudinal studies suggests that between 35% to 41.1% of pregnancies are unintended, including among women in HIV/AIDS care [2,7,8]. Comparable prevalence has been reported in neighboring countries, for example, Ethiopia and Sierra Leone have documented the prevalence of unintended pregnancy as 34.1% and 31.8%, respectively [3,4], while a recent Nigerian study reported a prevalence of 49.2% among women living with HIV [9]. The similarity of these findings across diverse settings highlights a regional pattern of persistently high unintended pregnancy driven by shared structural and social determinants.

Unintended pregnancy is influenced by several individual, socioeconomic, and health-system factors. At the individual level, younger maternal age, lower levels of education, lack of employment, increasing parity, lack of awareness of modern contraceptives, limited access to sexual and reproductive health information, and a history of adverse obstetric outcomes are often associated with unintended pregnancy [4,10,11]. Relationship-related factors like unmarried status, limited involvement of male partners, and poor communication regarding reproductive intentions have also been shown to increase the burden of unintended pregnancy [3,7,12].

Despite the magnitude of this burden and the high maternal mortality ratio of 189 per 100,000 live births in Uganda [13], context-specific evidence on unintended pregnancies from Southwestern Uganda remains scarce. This underscores the need for studies to document the burden of unintended pregnancy and its associated factors to inform targeted and equity-focused interventions. The objective of this secondary analysis was therefore to determine the prevalence of unintended pregnancy and document its associated factors among pregnant women at enrollment in the Support-Moms trial in Southwestern Uganda.

## Materials and methods

Study design and setting

This was a secondary analysis of the baseline data obtained from pregnant women enrolled in the Support-Moms study trial. The study was carried out in Mbarara City, Mbarara District, and Mitooma District, all located within Southwestern Uganda. These districts comprise both urban and rural settings and are served by both public and private health facilities. Overall, approximately 435,000 people reside within these districts [14].

Mbarara City is in the southwestern region of the country, approximately 270 km from the capital, Kampala. It hosts Mbarara Regional Referral Hospital, a tertiary institution that serves people from the 13 districts of the Ankole subregion, including Mitooma District. The other health facilities in the region that support pregnancy and childbirth are health centers (HC) II, III, IV, and general hospitals, both private and public health facilities, in accordance with the Ugandan health system structure. HC II and III provide basic obstetric emergency and newborn care, while hospitals and HC IV offer comprehensive obstetric and newborn care [15].

Study population

The study population consisted of pregnant women being recruited in the Support-Moms study project. The Support-Moms project was a controlled randomized trial that randomly assigned women into the control arm (receiving the standard of antenatal care) and the intervention arm that received health information messages and appointment reminders in the form of audio and Short Messaging Service (SMS) from the Support-Moms app. Eligible participants were pregnant women who were less than 20 weeks of gestation, who had access to a cell phone, were able to identify two social supporters, and had not started attending antenatal care. Any pregnant woman not meeting this whole criteria was not enrolled in the trial.

Sample size and sampling procedure

This study utilized the entire population of pregnant women enrolled in the Support-Moms study trial. A total of 824 from the three districts were enrolled in the study; however, six women had no clear information regarding pregnancy intention and were therefore not included in the final analysis.

Data collection procedures

Eligible women were identified by a team of Village Health Teams (VHTs) from communities and linked to the project, where they were re-assessed for eligibility by the research assistants. Data were collected at enrolment in the Support-Moms study project using interview-administered, structured questionnaires to obtain information on the study variables. A team of well-trained research assistants administered the questionnaire to all eligible mothers. This questionnaire was initially pretested to ensure the accuracy of the information being obtained.

Measurement of study variables

Dependent Variable

The outcome variable in this study was unintended pregnancy. Participants were asked about how they felt around the time when they conceived. The options included either (1) wanting to be pregnant sooner, (2) wanting to be pregnant later, (3) wanting to be pregnant then, and (4) not wanting to become pregnant then or at any time in the future. The outcome was taken as a binary variable. Women who wanted to become pregnant at the time they conceived or sooner were classified as having an intended pregnancy and coded 0, while those who wanted to become pregnant later or did not want to become pregnant at any time in the future were classified as having an unintended pregnancy and coded 1.

Independent Variables

Primary data were collected by interviewing participants. Food security was assessed using nine questions of the Household Food Insecurity Access Scale (HFIAS) that was freely accessed online [16] and was categorized as food secure, mild food insecure, moderate food insecure, and severe food insecure if they scored 0, 1-5, 6-13, and 14 points or more, respectively. The reported monthly income was categorized into two groups: those earning 150,000 Uganda shillings or less, the median income of females in Uganda, and those earning more than that [17]. Gravidity was categorized as primigravida (first pregnancy), multigravida (second to fourth pregnancy), and grand multigravida (fifth pregnancy or more).

Data management and analysis

Data were entered, cleaned, and analyzed using Stata version 19 (StataCorp LLC, College Station, TX). Descriptive analyses were conducted to summarize participant characteristics and study variables. Categorical variables were summarized using frequencies and percentages, while age was summarized using median and interquartile range since it was not normally distributed.

A modified Poisson regression analysis was performed to assess associations between independent variables and the study outcome. This model was preferred owing to the high prevalence obtained in this study, since logistic regression would overestimate the association in such a setting. Variables with a p-value < 0.20 at the bivariate level were included in the multivariable regression model to control for potential confounding and to identify factors independently associated with the outcome of interest. Multicollinearity was also assessed using the variance inflation factor (VIF). No variable had a VIF > 5. The p-value for goodness of fit was 1.000. Statistical significance was determined at a p-value < 0.05, and results were reported as prevalence ratios with the corresponding 95% confidence intervals.

## Results

A total of 818 participants were included in the final analysis. Most participants were from Mbarara District (56.4%). The median age of the study participants was 27 years (IQR: 23-32). The majority were aged 20-34 years (75.2%), married (96.6%), and had achieved a maximum Primary education (56.2%). The majority of the study participants were parous (63.4%) and reported a household income ≤150,000 Uganda Shillings; 9.5% of the study participants were HIV seropositive (Table 1).

**Table 1:** Baseline characteristics of study participants (N = 818)

Variable	Category	Frequency (n)	Percentage (%)
District	Mbarara City	235	28.7
	Mbarara District	461	56.4
	Mitooma District	122	14.9
Age (years)	Median age (IQR)	27	23-32
Age category	<20 years	56	6.8
	20-34 years	615	75.2
	≥35 years	147	18.0
Distance to health facility	<5 km	430	52.6
	≥5 km	388	47.4
Highest education	No formal education	20	2.4
	Primary	460	56.2
	Secondary	259	31.7
	Tertiary	79	9.7
Household income	≤150,000 UGX	512	62.6
	>150,000 UGX	306	37.4
Marital status	Married/lives with partner	790	96.6
	Unmarried	28	3.4
Food insecurity	Secure	78	9.5
	Mildly insecure	284	34.7
	Moderately insecure	353	43.2
	Severely insecure	103	12.6
Parity	Nulliparous	152	18.6
	Parous	519	63.4
	Grand multiparous	147	18.0
Gravidity	Primigravida	126	15.4
	Multigravida	502	61.4
	Grand multigravida	190	23.2
HIV status	Positive	78	9.5
	Negative	725	88.6
	Unknown	15	1.8
Knows partner’s desired number of children	No	370	45.2
	Yes	448	54.8

Prevalence of unintended pregnancy

**Figure 1:**
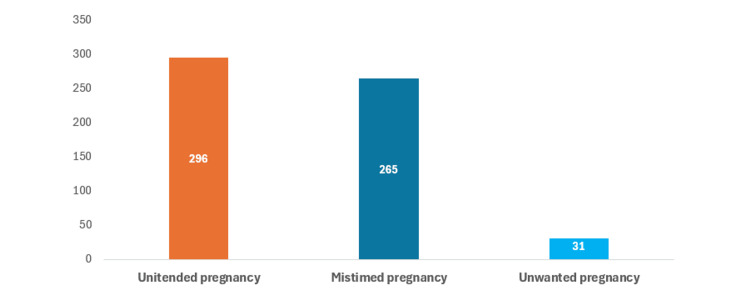
Bar graph showing the frequency of unintended pregnancy

Out of 818 women studied, 296 had an unintended pregnancy. The prevalence of unintended pregnancy in this group, therefore, was 36.2% (95% CI: 32.9-39.6). Most women had a mistimed pregnancy compared to an unwanted pregnancy (Figure 1).

Factors associated with unintended pregnancy

In the unadjusted analyses, unintended pregnancy was significantly associated with maternal age ≥35 years, unmarried status, moderate and severe food insecurity, increasing parity, grand multigravidity, and lack of awareness of the partner’s desired number of children (Table 2). However, in the multivariable modified Poisson regression model (Table 3), only three variables remained independently associated with unintended pregnancy. Women experiencing moderate food insecurity had a 55% higher prevalence of unintended pregnancy (adjusted prevalence ratio (aPR) = 1.55; 95% CI: 1.02-2.36), while those with severe food insecurity exhibited nearly twice the prevalence compared with food-secure women (aPR = 1.96; 95% CI: 1.25-3.06). Grand multigravida women (five or more lifetime pregnancies) also demonstrated a significantly increased prevalence relative to primigravida women (aPR = 1.64; 95% CI: 1.11-2.41). Furthermore, participants who were unaware of their partner’s preferred family size were more likely to experience unintended pregnancy (aPR = 1.22; 95% CI: 1.01-1.47).

**Table 2:** Univariate modified Poisson regression analysis of factors associated with unintended pregnancy in Southwestern Uganda cPR: crude prevalence ratio; Ref: reference category *p-value < 0.05

Variables	Unintended pregnancy		Unadjusted analysis cPR (95% CI)	p-value
	Yes (n = 296)	No (n = 522)		
	n (%)	n (%)		
Age category				
<20 years	15 (26.8)	41 (73.2)	0.78 (0.50-1.22)	0.278
20-34 years	211 (34.3)	404 (65.7)	Ref	-
35 years or older	70 (47.6)	77 (52.4)	1.39 (1.13-1.70)	0.001*
Distance to health facility				
<5 km	158 (36.7)	272 (63.3)	Ref	-
≥5 km	138 (35.6)	250 (64.4)	0.97 (0.81-1.16)	0.727
Highest level of education				
No formal education	9 (45.0)	11 (55.0)	1.19 (0.68-2.08)	0.553
Primary	171 (37.2)	289 (62.8)	0.98 (0.72-1.33)	0.891
Secondary	86 (33.2)	173 (66.8)	0.87 (0.63-1.22)	0.426
Tertiary	30 (38.0)	49 (62.0)	Ref	-
Household income categorized				
≤150,000 UGX	194 (37.9)	318 (62.1)	1.14 (0.94-1.38)	0.194
>150,000 UGX	102 (33.3)	204 (66.7)	Ref	-
Marital status				
Married/lives with partner	281 (35.6)	509 (64.4)	Ref	-
Unmarried	15 (53.6)	13 (46.4)	1.51 (1.05-2.15)	0.025*
Food insecurity				
Secure	18 (6.1)	60 (11.5)	Ref	-
Mildly insecure	91 (30.7)	193 (37.0)	1.39 (0.89-2.15)	0.143
Moderately insecure	135 (45.6)	218 (41.8)	1.66 (1.08-2.54)	0.020*
Severely insecure	52 (17.6)	51 (9.8)	2.19 (1.40-3.43)	0.001*
Parity				
Nulliparous	37 (24.3)	115 (75.7)	Ref	-
Parous	179 (34.5)	340 (65.5)	1.42 (1.04-1.92)	0.025*
Grandmultiparous	80 (54.4)	67 (45.6)	2.24 (1.63-3.07)	<0.001*
Gravidity				
Primigravida	33 (26.2)	93 (73.8)	0.78 (0.57-1.07)	0.122
Multigravida	169 (33.7)	333 (66.3)	Ref	-
Grand multigravida	94 (49.5)	96 (50.5)	1.47 (1.22-1.78)	<0.001*
HIV status				
Positive	28 (35.9)	50 (64.1)	Ref	-
Negative	263 (36.3)	462 (63.7)	1.01 (0.74-1.38)	0.947
Unknown	5 (33.3)	10 (66.7)	0.93 (0.43-2.02)	0.851
Knows partner’s desired number of children				
No	155 (41.9)	215 (58.1)	1.33 (1.11-1.60)	0.002*
Yes	141 (31.5)	307 (68.5)	Ref	-
Prior maternal complications during pregnancy				
No	265 (35.9)	474 (64.1)	Ref	-
Yes	31 (39.2)	48 (60.8)	1.09 (0.82-1.46)	0.544
Prior miscarriage				
No	237 (36.6)	411 (63.4)	Ref	-
Yes	59 (34.7)	111 (65.3)	0.95 (0.75-1.19)	0.655

**Table 3:** Multivariable modified Poisson regression analysis of factors associated with unintended pregnancy in Southwestern Uganda aPR: adjusted prevalence ratio; cPR: crude prevalence ratio; Ref: reference category *p-value < 0.05

Variables	Unadjusted analysis, cPR (95% CI)	p-value	Adjusted analysis, aPR (95% CI)	p-value
Age category				
<20 years	0.78 (0.50-1.22)	0.278	0.95 (0.58-1.56)	0.843
20-34 years	Ref	-	Ref	-
35 years or older	1.39 (1.13-1.70)	0.001*	1.08 (0.85-1.37)	0.507
Household income categorized				
≤150,000 UGX	1.14 (0.94-1.38)	0.194	1.03 (0.84-1.25)	0.798
>150,000 UGX	Ref	-	Ref	-
Marital status				
Married/lives with partner	Ref	-	Ref	-
Unmarried	1.51 (1.05-2.15)	0.025*	1.41 (0.96-2.08)	0.077
Food insecurity				
Secure	Ref	-	Ref	-
Mildly insecure	1.39 (0.89-2.15)	0.143	1.33 (0.86-2.04)	0.200
Moderately insecure	1.66 (1.08-2.54)	0.020*	1.55 (1.02-2.36)	0.041*
Severely insecure	2.19 (1.40-3.43)	0.001*	1.96 (1.25-3.06)	0.003*
Gravidity				
Primigravida	Ref	-	Ref	-
Multigravida	1.29 (0.94-1.77)	0.122	1.21 (0.85-1.72)	0.283
Grand multigravida	1.89 (1.36-2.62)	<0.001*	1.64 (1.11-2.41)	0.012*
Knows partner’s desired number of children				
No	1.33 (1.11-1.60)	0.002*	1.22 (1.01-1.47)	0.037*
Yes	Ref	-	Ref	-
Prior maternal complications during pregnancy				
No	Ref	-	Ref	-
Yes	1.09 (0.82-1.46)	0.544	1.09 (0.82-1.47)	0.544

## Discussion

In this study, the prevalence of unintended pregnancy among women in Southwestern Uganda was 36.2% (95% CI: 32.9-39.6), implying that about one-third of every pregnant woman has an unintended pregnancy. This is comparable to the prevalence of 34% reported in the 2022 Uganda Demographic Health Survey report and may be attributed to the low modern contraceptive prevalence rate (38%) in Uganda [13]. This prevalence is also comparable to what has been reported in other sub-Saharan countries such as Ghana [18], Cameroon [19], and Ethiopia [4]. This is possibly due to the shared sociodemographic determinants of pregnancy intention and the low contraceptive prevalence rates and high unmet need for family planning in both countries [20,21]. However, some studies have shown higher prevalence elsewhere. A study done in Tanzania showed a higher prevalence of unintended pregnancy (55.9%), with 32.5% mistimed and 13.4% unwanted [22], while a recent study conducted in Nigeria revealed a prevalence of 49.2% [9]. This variation may be attributed to differences in the study population; for example, the study in Nigeria focused on women living with HIV. There are other studies that have shown a lower prevalence, for example, 16.2% in Britain [23] and 17.2% in Sri Lanka, South Asia [24]. This is explained by the differences in the sociodemographic characteristics of women in developed countries.

The results of this study show that women who reported moderate food insecurity were more likely to have an unintended pregnancy (aPR = 1.55, 95% CI: 1.02-2.36), and those with severe food insecurity had a two-fold increase in the prevalence of unintended pregnancy (aPR = 1.96, 95% CI: 1.25-3.06). This is consistent with findings in the United States, where women of a lower socioeconomic status were more likely to have an unintended pregnancy [25]. Whereas it would be expected that such women experiencing food insecurity would embrace family planning practices to minimize their family size, on the contrary, it has been highlighted that women who experience food insecurity and other negative social determinants of health are less likely to use any contraceptive method despite not wanting to become pregnant [26]. Women with food insecurity may concurrently experience other social barriers, like low income or knowledge levels, that hinder their uptake of modern contraception. The study also demonstrates that women who had their fifth or more pregnancy were more likely to have an unintended pregnancy compared to primigravida women. This finding is consistent with results obtained from previous studies [27,28]. This may be because of inadequate family planning counselling among these women. Similar to study findings in Sierra Leone [3], women who did not know their partner’s desired number of children were 1.2 times more likely to have an unintended pregnancy compared to those who were aware of their partner’s desired number of children. It has also been noted that unintended pregnancy is more prevalent in families where females are the household heads compared to those headed by males [18]. Women who are not aware of their partner’s desired number of children may not fully take up family planning methods, resulting in mistimed or even unwanted pregnancies. This underscores the need for active male involvement in reproductive health services since they are key decision makers in many developing countries.

Strengths and limitations

This study had a very large sample size selected from three districts, both rural and urban settings, and is therefore representative of the population in Southwestern Uganda. This study, however, did not evaluate the number of women whose pregnancies were a result of contraceptive failure, which is an important contributor to unintended pregnancy. The study also depended on self-reported pregnancy intentions; there could have also been a degree of recall bias or social desirability bias, as many women may not want to disclose that the pregnancy was not desired due to cultural, social, and religious beliefs. This was a cross-sectional analysis and, therefore, lacks a temporal relationship for determining causality.

## Conclusions

This study found that at least one in every three pregnant women in Southwestern Uganda has an unintended pregnancy. Context-specific strategies should be put in place to address key drivers of this high burden, focusing on the most at-risk women, like those with food insecurity and other negative social determinants of health and grand multiparity. Male partners should be actively engaged in reproductive health activities to help in averting this high prevalence. There is a need to identify these women with unintended pregnancy and provide targeted support throughout pregnancy to optimize their outcomes.
